# Some Points of Preventive Treatment in the Diseases of Women

**Published:** 1897-04-10

**Authors:** Arthur E. Giles

**Affiliations:** Assistant Surgeon, Chelsea Hospital for Women


### SOME POINTS OF PREVENTIVE TREATMENT IN THE DISEASES OF WOMEN.

By Arthur E. Giles, M.D., B.Sc., M.R.C.P.Lond., F.R.C.S.Ed., Assistant Surgeon, Chelsea Hospital for Women.

It might be said with some measure of truth that, whereas surgery has saved its thousands, preventive medicine has saved its tens of thousands. This being so, the brilliant surgical results which have been achieved in the department of gynæcology (which, indeed, has become a branch of surgery rather than of medicine) should not cause us to forget that the work of prevention is still always with us.

In this communication I propose to consider some of the diseases of women from this point of view, not so much for the purpose of stating new propositions as to emphasise some that are well known. That there is a necessity for this seems evident, when we reflect how largely this class of disease might be prevented if the means of prevention were only borne in mind.

I shall first consider some of the disorders of menstruation. To what extent can painful menstruation, for instance, be prevented? Menstruation, being a physiological process, should be painless; yet, at a rough estimate, only one woman in every three is free from dysmenorrhœa; of the remainder, some suffer moderately, and can do their daily duties, but others suffer intensely, and are incapable of either work or pleasure, being confined to bed for a day or two every month. Women have become so accustomed to pain at their monthly periods that it has come to be regarded as a matter of course. But it should not be so, for in the earlier years after the onset of menstruation, the proportion of girls who suffer no pain is much greater; it is only later that pain becomes so common. This time of freedom from pain corresponds in the main to the time when girls are allowed to romp and run with their brothers, and when their life is in all respects more natural than it is when once they become "young women." When this time has arrived life is more artificial; the girl is expected to give up romping and active exercise: she must prepare for examinations, or she goes out to service or is apprenticed to a trade. This often means long hours of work, long standing, much confinement within doors, the "scamping" of meals, and sometimes considerable mental strain; so that at this, the first critical period of a woman's life, the whole nervous system is overtaxed, and the effects may last long. Now this is just the time when women often begin to menstruate painfully; and those who suffer most are precisely those of the class I have just described—pupil teachers and governesses, women studying for the higher examinations, shop assistants, servants, and those women who are of a highly-strung nervous temperament, or who lead a highly artificial life in society. Those who suffer least are girls brought up in the country, with its less hurried existence, and girls in the leisured classes who live simply.

It seems evident, therefore, that dysmenorrhœa might be to a great extent prevented by attention to general health and education. Short hours of work, especially of standing; plenty of outdoor exercise—tennis, boating, cycling, gymnastics, and walking for those who cannot afford these; regularity of meals, and food of the proper quantity—not the incessant tea and bread and butter with variations of pastry; the avoidance of over-exertion and prolonged fatigue; these are some of the principal things which require attention. These matters rest in the hands of parents, and they are important because it is not only the health of young girls that is at stake; it is, in addition, the health and happiness of wives and mothers that are to be. I wish to add a few words more on two points.

Firstly, the question of over-study. Probably the average girl can acquire as much learning as the average boy; but to do so she requires bodily health and strength equal to his. Now the boy and girl work under different conditions, which, if ignored, lead to disaster. Let girls pursue their study, but more leisurely; they will arrive at the same goal, but a little later. Physically and emotionally a girl arrives at womanhood earlier than a boy arrives at manhood; this necessitates a corresponding saving of energy in some direction, and the direction in which this economy of energy is to be sought is in intellectual activity.

Secondly, it should be impressed upon parents that premature emotional excitement is bad; sensational love novels should be avoided, and the "sex question" left dormant as long as possible. The idea that marriage is the only goal of a girl's existence should be discouraged, for while it may be true that in the *rôle* of wife and mother the average woman is seen at her best, the preparation for this position is best attained, not by directly aiming at it, but by the devolopment of physical health, by the training of the mind, by breadth of thought, and widening of interests.

I should like to point out here that when dysmenorrhœa exists, its treatment should not be confined to drugs and attempts at local relief, but should include those hygienic measures already indicated for its prevention.

Before leaving the subject of menstruation, I may dwell a moment on the importance of proper care at the menstrual periods, in order to avoid such complications as chills and pelvic inflammation. A careful exhortation from the medical man who may be consulted for other matters may lead a woman to understand that her system is more susceptible to harm at these times; and that the things to be specially avoided are getting the feet wet, or otherwise catching cold; bathing, when unaccustomed to it; exposure at night in evening dress; arduous exertion, such as dancing, long cycle rides, long day excursions or sight-seeing, especially in wet weather. Most women know all this, but the experience of their doctors shows that they too often ignore it. Considering what we know as to the evils so often following these practices, I urge that it is the duty of the profession to warn women against them; whereby much subsequent illness may be averted.

I pass on to consider briefly an important question, viz., gonorrhœa in women. Gonorrhœa is a preventable disease; yet it is responsible for an almost incredible amount of suffering and disease in women; principally, inflammation of the vagina and urethra; abcesses in the ducts of Bartholin, endometritis, suppurating disease of the Fallopian tubes and ovaries, and pelvic peritonitis. The results are chronic ill-health, dysmenorrhœa, sterility, and serious danger to life when the tubes and peritoneum are affected, necessitating in turn grave operations with their attendant risks. And all these things are preventable.

How? The question is, of course, largely one of public morality; to discourse on this is not my province, except to point out its results to the health of the community. It is to be feared that we do not always impress upon our patients that when a man exposes himself to gonorrhœal infection he is running the risk of placing in jeopardy the health or life of his present or future wife, and his own domestic happiness. Perhaps, with a few right-minded men, this consideration may have some weight. For the rest, it would seem to be the duty of the State to protect those who suffer through no fault of their own, namely, young wives and mothers. For it is they who suffer the heavy penalty; not men, in whom gonorrhœa is a comparatively mild disease. If any legislative measures can conduce to this protection by checking the spreading of a contagious disease, any opposing arguments are, it seems to me, futile; and if such measures as are at present proposed cannot accomplish this, the sooner others are introduced that can do so, the better for the public health.

Much may be done, however, in the early stages of gonorrhœa; and I would urge that prompt treatment, carried out while the disease is still confined to the urethra, vagina, and even to the endometrium, would avert many of the most serious disasters—those, namely, which are associated with implication of the Fallopian tubes and peritoneum; for it is when these are involved that the patient becomes a chronic invalid; that sterility is produced; that married life becomes impossible; that the woman's life is in danger: and that the only hope of saving health and life lies in the performance of operations which must be considered as among the most difficult and serious of surgical procedures. All surgeons who have had much practical acquaintance with operations for pyosalpinx, with their technical difficulties due to adhesions, and their dangers of septicæmia, will testify to the truth of these observations.

The best plan of treatment in the early stages of gonorrhœa, is to place the patient under an anæsthetic, to irrigate the vagina thoroughly with a 1 in 1,000 solution of biniodide of mercury, to swab out the uterine cavity with iodised phenol on a Playfair's probe, and to leave in the vagina a glycerine tampon covered with iodoform. Antiseptic douches are then given twice daily for a week and a glycerine tampon inserted each night, to be removed the next morning. Constitutional treatment, with diuretics, must be given at the same time.

The diseases of women are naturally closely associated with the function of child-bearing, and so I may next and suitably consider the question of the prevention of those diseases which result from miscarriages.

As to criminal abortions, without entering into the ethical aspects of the matter, I may properly point out that besides the great danger to life which attends them, partly from injury to important organs, and partly from blood-poisoning, these rash and criminal attempts are, no doubt, often the origin of chronic disease; for the miscarriage is often induced unskilfully, the necessary secrecy prevents the exercise of proper after-care, and septic troubles are apt to arise which may be the unsuspected cause of many morbid conditions met with later.

It is of the disorders and diseases following accidental miscarriage that I wish more especially to speak. The question is important because of its frequency; comparatively few women pass through their child-bearing period without one or more miscarriages.

The first danger is hæmorrhage. It is by no means uncommon to find a condition of prolonged ill-health date back to a profuse loss of blood at a neglected miscarriage. All this may be prevented or checked by proper treatment at the time, the patient being kept in bed under strict supervision; whilst any serious hæmorrhage should be the signal for at once clearing out the uterus thoroughly under an anæsthetic. This will avert immediate risk, and save the patient from much subsequent trouble.

The second risk is blood-poisoning; which can always be avoided by proper antiseptic precautions.

The third is retention of products of conception. Special care should be taken to see that the whole of the placenta comes away; otherwise a small portion may remain and lead to long-continued hæmorrhage. It may do more: it readily becomes the seat of decomposition, and may be followed by chronic endometritis and subinvolution, or by septicæmia. Briefly, when a miscarriage occurs, as much care is required as if the woman were being delivered of a child at full time. I may go further, and give it as my opinion that however much it may be maintained that nature or a midwife suffices for an ordinary confinement, every miscarriage requires a doctor, with all the skill and care which he can exercise.

Unfortunately, women do not seem to think so. In a large number of cases they struggle through these accidents with the smallest possible amount of rest and attention, and the result is that miscarriages, perhaps even more than confinements, figure among the causes of diseases of women, diseases which certainly could be prevented by proper care at the proper time.

